# Filling gaps of genome scaffolds via probabilistic searching optical maps against assembly graph

**DOI:** 10.1186/s12859-021-04448-2

**Published:** 2021-10-30

**Authors:** Bin Huang, Guozheng Wei, Bing Wang, Fusong Ju, Yi Zhong, Zhuozheng Shi, Shiwei Sun, Dongbo Bu

**Affiliations:** 1 Key Lab of Intelligent Information Processing, Big-Data Academy, Institute of Computing Technology, Chinese Academy of Sciences, Beijing, 100190 China; 2 Institute of Biology, University of Chinese Academy of Sciences, Beijing, 100049 China; 3 School of Computer Science, University of Washington, Seattle, 98195 USA; 4 Department of Computer Science and Engineering, University of California, San Diego, La Jolla, 92093 USA; 5 Zhongke Big Data Academy, Zhengzhou, 450046 Henan China

**Keywords:** Genome assembly, Gap filling, Scaffolding, Optical maps, Probabilistic search

## Abstract

**Background:**

Optical maps record locations of specific enzyme recognition sites within long genome fragments. This long-distance information enables aligning genome assembly contigs onto optical maps and ordering contigs into scaffolds. The generated scaffolds, however, often contain a large amount of gaps. To fill these gaps, a feasible way is to search genome assembly graph for the best-matching contig paths that connect boundary contigs of gaps. The combination of searching and evaluation procedures might be “searching followed by evaluation”, which is infeasible for long gaps, or “searching by evaluation”, which heavily relies on heuristics and thus usually yields unreliable contig paths.

**Results:**

We here report an accurate and efficient approach to filling gaps of genome scaffolds with aids of optical maps. Using simulated data from 12 species and real data from 3 species, we demonstrate the successful application of our approach in gap filling with improved accuracy and completeness of genome scaffolds.

**Conclusion:**

Our approach applies a sequential Bayesian updating technique to measure the similarity between optical maps and candidate contig paths. Using this similarity to guide path searching, our approach achieves higher accuracy than the existing “searching by evaluation” strategy that relies on heuristics. Furthermore, unlike the “searching followed by evaluation” strategy enumerating all possible paths, our approach prunes the unlikely sub-paths and extends the highly-probable ones only, thus significantly increasing searching efficiency.

**Supplementary Information:**

The online version contains supplementary material available at 10.1186/s12859-021-04448-2.

## Background

Genome assembly aims to reconstruct genomes from sequencing reads, and thus plays important roles in various downstream studies, including identification of genes and genome structure variations. Most of the existing assembly methods first organize sequencing reads into a graph, say de Bruijn graph or overlap graph, and then attempt to find a path in the graph to restore the original genome sequence [1]. However, the genome repeats longer than sequencing reads always create ambiguities in path finding, making assembly approaches yield only separate paths (called *contigs*) rather than the complete genomes [2]. The longer reads by third generation sequencing [3, 4], and long-distance linking information by pair-end, mate-pair, or mapping technologies, will definitely help genome assembly methods to resolve the ambiguities incurred by repeats [5]. The study [6] provides an elaborated review on the methodological progresses and perspectives in the integration of short-range and long-range information for improving assembly contiguity.

Among the technologies that provide long-distance information across repeats, optical mapping has its unique advantage in measuring long genome fragments. For example, the BioNano Saphyr platform can measure genome fragment up to 2 megabases [7]. Unlike genome sequencing technologies, optical maps record locations of specific enzyme recognition sites, say GCTCTTC and GAAGAGC for enzyme BspQI, along a genome. By identifying these sites from contigs, we can easily align contigs onto optical maps, and then order them into *scaffolds* [8]. However, the short contigs that contain insufficient enzyme recognition sites usually cannot be reliably aligned onto optical maps, thus creating a variety of gaps in scaffolds and making them far from complete genomes. Filling these gaps with nucleotide sequence will considerably improve the completeness of genome assembly.

A great variety of approaches have been proposed for filling gaps directly using sequencing reads, including SOAPdenovo [9], GapFiller [10], GMCloser [11], PBJelly [12] and LR_Gapcloser [13]. These approaches, however, are infeasible for filling gaps of the scaffolds obtained via optical maps since these gaps are often much longer than sequencing reads. To fill these large gaps, Nagarajan *et al.* proposed to use contig paths in assembly graph instead of the short sequencing reads [14]. Here, assembly graphs refer to the product of assembling sequencing reads using graph theory, which contains contigs as nodes and connections among them as edges.

Recent progresses to improve assembly contiguity also include Bionano solve pipeline, BiSCoT [15], and Novo&Stitch [16]. Briefly speaking, Bionano solve pipeline uses a module called “Hybrid Scaffold”, which sets the identified gaps with N-base rather than filling them using genome sequence. BiSCoT aims to resolve the N’s gap between contigs inserted by Bionano scaffolding through merging two contings that share a genomic region. Novo&Stitch proposes a novel method that uses optical maps for accurate assembly reconciliation.

To fill gaps, we can choose a contig path that connect two boundary contigs of a gap, and then uses the corresponding nucleotide sequences. Thus, the successful gap-filling relies on two steps: (1) searching contig paths in assembly graph, and (2) evaluating the consistency between contig paths and optical map of the gaps of interest [17–19]. The two steps, i.e., searching and evaluating contigs paths, can be combined in various ways. For example, OMACC [17] employs the “searching followed by evaluation” strategy. Specifically, for the two boundary contigs of a gap, OMACC first searches assembly graph for all possible contig paths to connect them. Next, OMACC evaluates each possible contig path in terms of the difference between path length and gap size and selects the best path to fill the gap. By rescaling optical maps and estimating the number of repeat copies within gaps, OMACC achieved better accuracy than the previous studies [14, 17].

In contrast to OMACC, AGORA employs the “searching by evaluation” strategy [18]. That is, AGORA uses a modified depth-first search (DFS) to identify the most likely contig path. At each search step, AGORA selects an edge to extend the current sub-path according to several heuristics, say the decreasing order of edges, the consistency between this edge’s *in silico* map to the experimental optical maps. AGORA uses the first found contig path to fill a gap. These heuristics could greatly improve searching efficiency; however, they might also lead to potential errors in genome reconstruction.

In summary, the “searching followed by evaluation” strategy has high accuracy but low efficiency, whereas the “searching by evaluation” strategy has high efficiency but low accuracy. Thus, the tradeoff between accuracy and efficiency remains a challenging task.

In this study, we propose an accurate and efficient approach to gap filling. Unlike the existing “searching by evaluation” methods heavily relying on heuristics, our approach uses a stochastic model to calculate the similarity between optical maps and contig paths. Using the calculated similarity to guide path-finding, our approach achieves higher accuracy than the existing approaches using heuristics. In addition, unlike the “searching followed by evaluation” methods, our approach maintains only a small set of highly probable sub-paths and prunes the unlikely ones, thus significantly improving efficiency.

We evaluated nanoGapFiller on simulated optical maps of 12 species and real optical maps of 3 species. On the simulated data sets, nanoGapFiller fills the gaps with high accuracy in minutes. Moreover, nanoGapFiller always fills more gaps than OMACC. On real data sets, OMACC cannot fill any gap, while nanoGapFiller successfully fills all of the identified gaps. We also showed that our pruning strategy could significantly reduce running time without sacrificing accuracy. Thus, nanoGapFiller should benefit various downstream genomic studies by improving the completeness of genome reconstruction with aid of optical maps.

## Results

### Experiment setting and evaluation criteria

We evaluated accuracy and efficiency of nanoGapFiller on simulated optical maps of 12 species and real optical maps of 3 species. The real optical maps were acquired using BioNano Iris platform: For *E. coli*, *P. putida* and *S. coelicolor*, the number of optical maps are 8644, 15000 and 17422 respectively, and the coverage are 336, 435 and 354 respectively. The simulated optical maps were generated using an in-house simulator to extract enzyme recognition sites from reference genomes. We also applied another simulator OMsim [20] that adopts different error model from our in-house simulator.

The gaps of scaffolds were identified as follows: Using the reference genome of a species, we first generated simulated next-generation sequence (NGS) reads using read simulator ART [21], and then assembled these reads into assembly graph using genome assembler SPAdes [22]. Each simulated datasets has read length of 150, coverage of 50. Next, we aligned contigs onto optical maps, and further ordered the contigs into scaffolds according to the alignments. Finally, the unaligned parts of scaffolds were identified as gaps. To make thorough evaluation, we adopted two types of alignment methods: (1) SOMA2 used by OMACC [23], and (2) refAligner used by BioNano Solve package. Compared with SOMA2, refAligner generally reports fewer alignments with higher precision, and thus generates longer and more accurate gaps.

We assessed the quality of gap filling through calculating two levels of similarity between gap filling results and the corresponding regions in reference sequences:

1. *Contig path similarity (CPS)*: the number of contigs shared by the filled gaps and the real contig paths in reference genomes.
2. *Nucleotide sequence similarity (NSS)*: we further calculated the base-level similarity $NSS = 2 \times L_c / (L_r + L_f)$, where $L_f$ and $L_r$ denote the length of gap filling results and corresponding reference sequence, respectively, and $L_c$ denotes the longest common string between them.

In the study, we compared nanoGapFiller with the state-of-the-art software OMACC. We did not perform comparison with AGORA since it is now out of maintenance.

### Evaluating accuracy of gap filling

Table 1 shows the accuracy of gap filling results on the *E. coli* genome. As shown in this table, nanoGapFiller successfully filled all of these 23 gaps with NSS over 99%. In contrast, OMACC could only fill 11 out of the 23 gaps and failed to fill the long gaps with over 15 contigs. Even for these 11 gaps, OMACC’s quality is not always high. For example, for the gap 252216r-252238, its reference sequence consists of 7 contigs of 226 nt; however, OMACC filled this gap with 31 contigs of 1546 nt, which has considerably low similarity with the reference sequence (NSS: $25.51\%$). On the other 11 species, the gap filling results again suggest the superiority of nanoGapFiller in terms of accuracy and coverage (Additional file 1: Tables 1–11 and Additional file 1: Fig. 1). As shown in Additional file 1: Tables 14, 15, and 16, nanoGapFiller also shows better performance than Novo&Stitch.

**Fig. 1:**
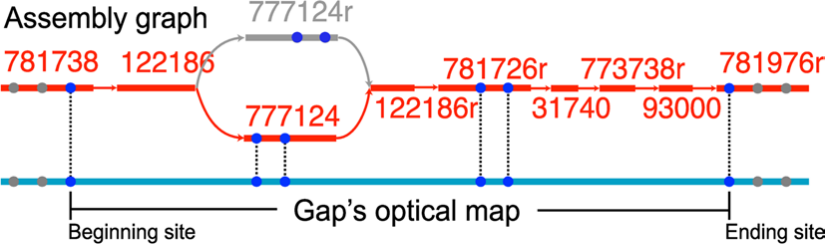
A case study of gap filling using OMACC and nanoGapFiller. For the the gap 781738-781976r of *S. coelicolor*, there are two contig paths connecting the beginning site and ending site: one path contains the contig 777124 while the other path contains its reverse complement 777124r. OMACC explores the distance between the beginning site and ending site only, and thus cannot identify which path matches better with the corresponding optical map. In contrast, nanoGapFiller utilizes the enzyme recognition sites in the intermediate contig 777124 and 781726r and thus correctly identified the contig path that fills the gap (shown in red)

As a concrete example, we showed in Fig. 1 the filling process of the gap 781738-781976r of *S. coelicolor*. There are two contig paths connecting the beginning site and ending site of the gap: one path contains the contig 777124 while the other path contains its reverse complement 777124r. OMACC explores the distance between the beginning site and ending site only, and thus cannot identify which path matches better with the corresponding optical map. In contrast, nanoGapFiller utilizes the enzyme recognition sites in the intermediate contigs 777124 and 781726r. Specifically, both 777124 and its reverse complement 777124r contain two sites; however, the locations of these sites differ greatly in the two contigs. nanoGapFiller exploited this difference and thus correctly identified the contig path that fills the gap.

**Table 1:** Filling the gaps identified using simulated optical maps of *E. coli* genome

Gap	Reference sequence		OMACC				nanoGapFiller			
	#contigs	#bases	#contigs	#bases	CPS	NSS (%)	#contigs	#bases	CPS	NSS (%)
252228–252408r	4	11	4	11	4	100	4	11	4	100
252424r–252466	4	12	46	3316	4	0.72	4	12	4	100
252292–252268r	5	645	5	645	5	100	5	645	5	100
252410r–252292	5	10,858	5	10,858	5	100	5	10,858	5	100
252208–252300	5	199	7	367	5	70.32	5	199	5	100
252538r–252526	6	1256	–	–	–	–	6	1256	6	100
252216r–252238	7	226	31	1546	7	25.51	7	226	7	100
252238–252228	7	1259	7	1259	7	100	7	1259	7	100
251622r–252244	9	30,034	17	30,482	9	99.26	9	30,034	9	100
252244–252386r	10	10,424	10	10,424	10	100	10	10,424	10	100
251936r–252510	14	18,913	–	–	–	–	14	18,913	12	99.86
252408r–252538r	14	20,922	14	20,922	14	100	14	20,922	14	100
252268r–252316	15	3339	19	3701	15	94.86	15	3339	15	100
252300–252312	15	5127	–	–	–	–	15	5127	11	98.93
252132–252226	18	7171	–	–	–	–	18	7171	18	100
252180–252410r	23	22,821	–	–	–	–	24	22,821	20	99.40
252316–252290	23	79,025	–	–	–	–	23	79,025	22	99.99
252312–252424r	24	5431	–	–	–	–	25	5431	23	99.96
252466–252252	25	13,442	–	–	–	–	26	13,442	21	99.97
252226–252216r	42	156,131	–	–	–	–	43	156,131	33	99.93
252486–252208	47	83,834	–	–	–	–	47	83,834	41	99.92
252148–252180	56	50,774	–	–	–	–	59	50,774	44	99.20
252252–252310r	59	118,754	–	–	–	–	60	118,754	53	100

Alignment method: SOMA2. Here, the symbol ‘–’ represents the failure of OMACC

**Table 2:** Filling the gaps identified using real optical maps of *E. coli* genome

Gap	Reference sequence		OMACC				nanoGapFiller			
	#contigs	#bases	#contigs	#bases	CPS	NSS (%)	#contigs	#bases	CPS	NSS (%)
252486r–252036r	3	954	3	954	3	100	3	954	3	100
252408–252228r	4	11	4	11	4	100	4	11	4	100
252526r–252538	6	1256	6	1256	6	100	6	1256	6	100
252032–252526r	11	1127	11	1127	11	100	11	1127	11	100
252538–252408	14	20,922	14	20,922	14	100	14	20,922	14	100
252312r–252300r	15	5127	–	–	–	–	15	5127	13	99.06
252290r–251900r	21	18,454	–	–	–	–	31	19,755	20	96.59
252252r–252466r	26	13,442	–	–	–	–	37	13,877	21	97.69
252036r–252032	26	36,757	–	–	–	–	38	37,430	19	99.08

Alignment method: SOMA2. Here, the symbol ‘–’ represents the failure of OMACC

**Fig. 2:**
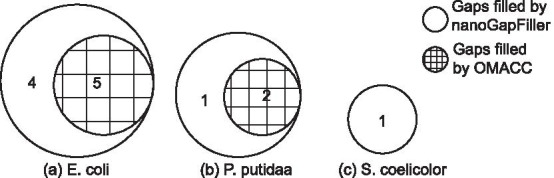
Venn graphs of the gaps filled by nanoGapFiller and OMACC for **a** *E. coli*, **b** *P. putida*, and **c** *S. coelicolor*. Here, the gaps are identified using real optical maps with alignment method SOMA2

**Table 3:** Filling the gaps identified using real optical maps of *E. coli*, *S. coelicolor*, and *P. putida* genomes

Species	Gap	Reference sequence		nanoGapFiller		
		#contigs	#bases	#contigs	CPS	NSS (%)
*E. coli*	252196–252226	17	51,810	20	17	99.66
*E. coli*	252486r–252526r	35	105,652	38	27	99.79
*E. coli*	252510r–252292r	93	600,523	107	86	99.84
*E. coli*	252312r–252486r	108	184,041	65	31	87.54
*S. coelicolor*	781738–781976r	9	88,470	9	9	100
*S. coelicolor*	781976r–781848r	18	66,018	18	18	100
*P. putida*	443944r–443818	95	875,288	96	83	99.88

Alignment method: refAligner

**Table 4:** Running time (in seconds) of nanoGapFiller for filling the gaps of 12 species

Species	#Contigs in assembly graph	#Sites in site graph	#Edges in site graph	#Filled gaps	Total length of gaps (nt)	Running time (s)
*S. ynec*	250	774	1905	11	181,832	5.24
*S. coelicolor*	926	3,532	8160	17	478,321	25.95
*S. agal*	204	546	5554	16	463,622	8.04
*P. syringae*	1492	2176	51,343	25	732,210	371.05
*P. putida*	752	2190	15,759	9	2856,292	188.39
*N. farcinica*	388	924	2192	19	639,552	10.15
*E. coli*	734	1348	40,858	23	922,021	88.08
*E. carotovora*	622	1454	2636	19	586,169	9.42
*C. hutchinsonii*	596	990	5105	25	895,179	12.24
*B. pseudomallei*	390	2144	2662	8	175,737	3.83
*B. japonicum*	992	3524	26,014	29	1021,611	1721.27
*A. vari*	870	474	135,667	17	4917,178	71,067.07

Here, the gaps are identified using simulated optical maps with alignment method SOMA2. CPU: AMD Opteron 6344; OS: Ubuntu 16.04; Python version: 3.6.7

**Table 5:** Running time (in seconds) of nanoGapFiller at different settings of pruning threshold MinimalMatchingProbability

Dataset	Alignment method	MinimalMatchingProbability			
		0 (no pruning)	$10^{-8}$	$10^{-5}$ (default)	$10^{-2}$
Simulated optical maps	SOMA2	2123	90	88	53
Real optical maps	SOMA2	46	13	11	13
Real optical maps	refAligner	5953	1620	1227	941

Here, the gaps are identified using both simulated and real optical maps of *E. coli* species. CPU: AMD Opteron 6344; OS: Ubuntu 16.04; Python version: 3.6.7

**Table 6:** The quality of filled gaps reported by nanoGapFiller at different settings of pruning threshold MinimalMatchingProbability

Species	Gap	MinimalMatchingProbability			
		0 (%)	$10^{-8}$ (%)	$10^{-5}$ (default) (%)	$10^{-2}$ (%)
*E. coli*	252312r–252486r	87.22	87.22	87.22	87.22
*E. coli*	252486r–252526r	99.68	99.68	99.68	99.68
*E. coli*	252510r–252292r	98.87	98.87	98.87	96.55
*E. coli*	252196–252226	99.66	99.66	99.66	99.66
*S. coelicolor*	781738–781976r	100.00	100.00	100.00	100.00
*S. coelicolor*	781976r–781848r	100.00	100.00	100.00	100.00
*P. putida*	443944r–443818	99.88	99.88	99.88	99.88

Here the gaps are identified using real optical maps and alignment method refAligner. The quality is measured using base-level similarity (NSS) between the filled gaps and the corresponding reference genome sequence

**Table 7:** Genome completeness improvement after filling gaps using OMACC and nanoGapFiller on 12 species

Species	Scaffold N50 (nt)		
	Before gap filling	Filling using OMACC	Filling using nanoGapFiller
*A. vari*	64,556	78,980	7,589,442
*B. japonicum*	143,477	290,961	1,830,875
*B. pseudomallei*	86,778	99,967	113,112
*C. hutchinsonii*	129,478	212,390	1,935,216
*E. carotovora*	71,290	100,730	680,365
*E. coli*	78,648	140,985	1,222,147
*N. farcinica*	176,628	846,096	5,627,295
*P. putida*	127,879	127,879	4,873,348
*P. syringae*	79,967	90,066	366,420
*S. agal*	71,533	1,399,536	2,406,989
*S. coelicolor*	108,454	120,270	213,619
*S. ynec*	175,767	300,280	1,774,968

Here, the gaps are identified using simulated optical maps and alignment method SOMA2

**Table 8:** Genome completeness improvement after filling gaps using OMACC and nanoGapFiller on 3 species

Species	Scaffold N50 (nt)		
	Before gap filling	Filling using OMACC	Filling using nanoGapFiller
*E. coli*	78,648	107,371	124,003
*P. putida*	127,879	154,105	154,105
*S. coelicolor*	108,454	108,454	108,454

Here, the gaps are identified using real optical maps and alignment method SOMA2

**Table 9:** Genome completeness improvement after filling gaps using OMACC and nanoGapFiller on 3 species

Species	Scaffold N50 (nt)		
	Before gap filling	Filling using OMACC	Filling using nanoGapFiller
*E. coli*	78,648	78,648	133,054
*P. putida*	127,879	127,879	154,105
*S. coelicolor*	108,454	108,454	109,573

Here, the gaps are identified using real optical maps and alignment method refAligner

We further investigated the accuracy of nanoGapFiller on real optical maps of three species (Table 2, Additional file 1: Tables 12, 13). As shown in Table 2, only 9 gaps were identified when using SOMA2 as alignment method on *E. coli* species, which is less than those identified on the simulated optical maps. OMACC successfully filled 5 out of 9 gaps but failed at the other 4 gaps with over 15 contigs. In contrast, nanoGapFiller filled all of these 9 gaps with considerably high accuracy (NSS over 96%). Venn graphs suggest the superiority of nanoGapFiller in terms of coverage on these 3 species (Fig. 2).

When using refAligner to align contigs onto optical maps, only 4, 2, and 1 gaps were identified for *E. coli*, *S. coelicolor*, and *P. putida* species, respectively (Table 3). The longest gap has 875Knt. OMACC failed at all of these 7 gaps. In contrast, with only one exception (252312r-252486r), nanoGapFiller successfully filled all gaps with nucleotide sequence highly similar to the reference genome (NSS over 99%). We also evaluate nanoGapFiller using OMBlast [24] as alignment tool. As shown in Additional file 1: Table 18, a total of 23 gaps are identified. Despite that these gaps are different from the gaps when using SOMA2 as alignment tool (Additional file 1: Table 17), nanoGapFiller can still successfully fill these gaps with significant gap filling performance (NSS over 97%).

In addition to evaluating our approach on simulated optical maps generated by in-house simulator, we also repeated the evaluation process on the optical maps generated using OMsim that adopts a different error model. As shown in Additional file 1: Table 19, a total of 8 gaps were identified and for 7 out of the 8 gaps, nanoGapFiller achieves accurate gap filling with NSS exceeding 97%. These results clearly demonstrate that even using simulators with different error models, nanoGapFiller can still reliably accomplish gap filling.

Hi-C scaffolding [25, 26] is a promising approach that bridge and order contigs through exploiting the contact frequencies between pairs of loci [27]. Here, we compare nanoGapFiller with 3D-DNA [28], a software for Hi-C scaffolding, using the Hi-C data downloaded from NCBI GEO (GSM2870416, GSM2870417) [29]. As shown Additional file 1: Table 20, 3D-DNA achieves largest contig, total length and N50 of 4375178 bp, 4637496 bp and 4375178 bp, respectively, which is higher than that of nanoGapFiller (894614 bp, 4597570 bp and 785645 bp, respectively). However, 3D-DNA simply fills the gaps with N-bases rather than genome sequence. Thus, we further calculate NA50 where the contigs are replaced with the blocks that can be aligned to the reference. nanoGapFiller achieves an NA50 of 785645 bp, which is much higher than 3D-DNA (438708 bp).

### Evaluating efficiency of gap filling

In this section, we analyzed the running time of nanoGapFiller. Theoretically, the probabilistic search procedure takes *O*(*m*|*E*|) times, where *m* denotes the number of sites in gaps, and |*E*| denotes the number of edges in the site graph. As shown in Table 4, for 11 out of 12 species, nanoGapFiller takes only minutes on an ordinary personal computer. For *A. vari*, the gaps contain 4,917,178 nt in total, and the site graph contains 135,667 edges, thus leading to an expensive time cost (71,067.07 s).

One of the key points of our approach is pruning the unlikely sub-paths when the matching probability is below MinimalMatchingProbability. As shown in Table 5, nanoGapFiller uses 2123 s when no pruning is applied; in contrast, it takes only 88 s when setting MinimalMatchingProbability as $10^{-5}$. On the other side, the gap filling results nearly never change at different settings of the pruning threshold MinimalMatchingProbability (Table 6). Together, these observations clearly suggest that our approach perfectly balances the accuracy and efficiency in gap filling.

### Improvement of completeness of genome scaffolds

Finally we examined the improvement of completeness of genome scaffolds with gaps filled. As shown in Table 7, before filling gaps, the contigs are relatively short for *A. vari* species (N50: 64,556 nt). After filling the gaps using OMACC, the scaffold N50 increased to 78,980 nt. In contrast, after filling gaps using nanoGapFiller, the scaffold N50 increased to 7,589,422 nt, which is remarkably longer than that was reported using OMACC. We could observe similar results on other 11 species and real datasets (Tables 8 and 9).

To acquire more detailed evaluations, we have further applied Quast [30] to calculate multiple metrics of the assembly results (Additional file 1: Tables 14, 15, and 16).

## Discussion

In this study, we present an efficient and effective approach for fill gaps of scaffolds with aid of optical maps. Using probabilistic search, our approach perfectly balances the accuracy and efficiency of gap filling. The performance of our approach has been clearly demonstrated by the results on a variety of species using both simulated and real optical maps.

For large genome, the current version of nanoGapFiller suffers from the limitation that it generates a large size site graph which poses high memory requirement. How to improve our approach to reduce memory requirement remains one of the future studies.

## Conclusion

In conclusion, nanoGapFiller can effectively improve the contiguity of genome assembly. We expect that our approach, with potential extensions, can greatly facilitate improving completeness of genome assembly.

## Methods

### Notations

In genome sequencing and assembly, a *contig* refers to a contiguous nucleotide sequence resulting from assembly of sequencing reads, whereas a *scaffold* refers to a series of contigs separated by gaps of estimated length.

Unlike genome sequence reads, an *optical map* records locations of specific enzyme recognition sites along a molecule of DNA. Specifically, for a molecule consisting of *n* recognition sites $s_1, s_2, \cdots , s_n$, optical maps count the number of nucleotide bases between $s_i$ and $s_{i+1}$ for $1\le i \le n-1$, which is denoted as $d(s_i, s_{i+1})$. For example, the molecule GCTCTTCACGCTCTTCACTGCTCTTC has three appearances of the enzyme recognition site GCTCTTC, and the corresponding optical map records the distance between these sites, i.e., $d(s_1, s_2) = 9$, $d(s_2, s_3) = 10$. In the study, we write a site sequence as $s_b\cdots s_e$, where the symbol ‘$\cdots$’ represents the intermediate sites, and $s_b$ and $s_e$ denotes the beginning and ending site of the sequence, respectively.

**Fig. 3:**
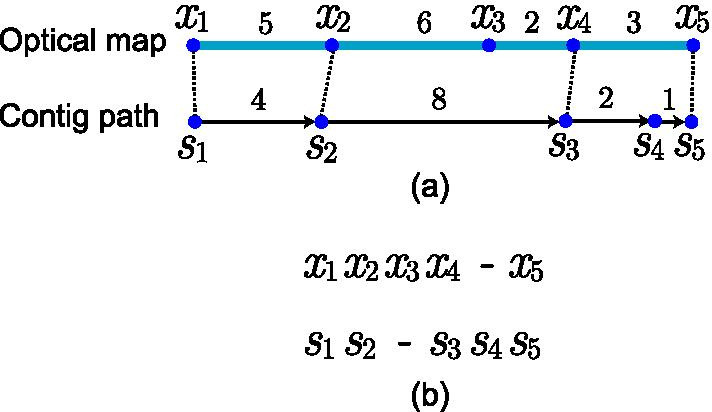
An example of the alignment between optical map and contig path. **a** An alignment corresponding to the generating of $x_1\cdots x_5$ from $s_1\cdots s_5$, where $<x_1, s_1>$, $<x_2, s_2>$, $<x_4, s_3>$ and $<x_5, s_5>$ are matching sites, while $s_4$ is a missing site and $x_3$ is a false-positive site. **b** The formal description of the alignment, where the symbol ‘–’ represents an Insertion or Deletion

Most genome assembly approaches utilize graph theory to guide assembly and finally generate an *assembly graph*, which contains contigs as nodes and connections among them as edges. To accelerate searching optical maps against assembly graph, we transform assembly graph into *site graph* as follows: from the component contigs of the assembly graph, we first identify all appearances of the enzyme recognition sites. Next, we use these sites as nodes, and connect the neighboring sites with edges. Here, we say two sites are neighbors if one site can be directly reached from another one by following a contig path in the assembly graph. Each edge in a site graph is associated with a distance to represent the number of nucleotide bases between the two corresponding sites (Fig. 3).

### Workflow of nanoGapFiller

**Fig. 4:**
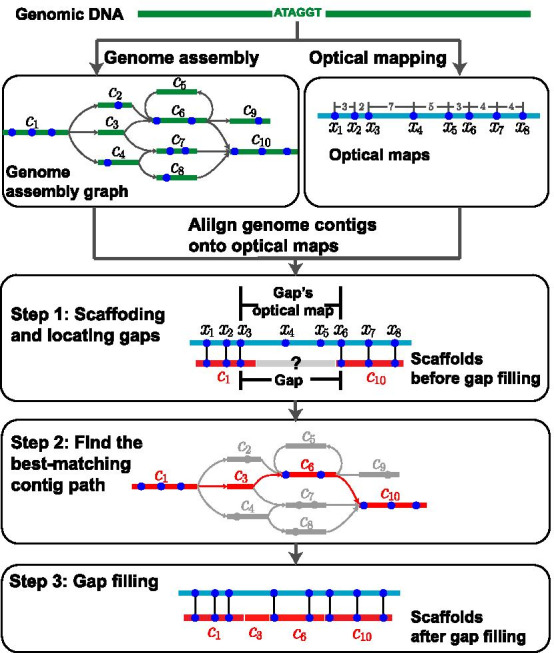
Overall pipeline of nanoGapFiller. Step 1. Initially, nanoGapFiller aligns genome assembly contigs onto optical maps. The aligned contigs are further connected into scaffolds according to their order in the alignment. Note that some regions of optical maps often fail to align with any contig, thus forming gaps in scaffolds. Here, we identified a gap with site sequence $x_3 x_4 x_5 x_6$. Step 2. To fill this gap, nanoGapFiller searches in assembly graph the contig path (shown in red) that matches best with the site sequence $x_3x_4x_5x_6$. Step 3. nanoGapFiller fills the gap with the nucleotide sequence of the best-matching contig path $c_1c_3c_6c_{10}$

nanoGapFiller takes experimental optical maps and genome assembly graph as input and generates scaffolds with gaps filled as output. As shown in Fig. 4, the workflow of nanoGapFiller mainly consists of the following three steps:

1. *Scaffolding and locating gaps:* Initially, nanoGapFiller aligns genome assembly contigs onto optical maps. The aligned contigs are further connected into scaffolds according to their order in the alignment. Note that some regions of optical maps often fail to align with any contig, thus forming gaps in scaffolds. These gaps, represented as Ns rather than normal nucleotide bases A/C/T/G, might be thousands of bases long.
  For each gap, we record three features, namely, beginning site, ending site, and the site sequence excerpted from the corresponding unaligned region of an optical map. Take the gap shown in Fig. 4 as an example, its beginning site and ending site are $x_3$ and $x_6$, respectively, and its site sequence is $x_3 x_4 x_5 x_6$.
2. *Finding the contig path matching best with gaps:* To fill a gap of scaffolds, nanoGapFiller searches assembly graph for the contig path that matches best with the site sequence of the gap. For this aim, nanoGapFiller uses a stochastic model to measure the similarity between a site sequence and any possible contig path, and then uses the probabilistic search technique to efficiently identify the contig path with the highest similarity. The details of the stochastic model and the probabilistic search technique will be described in later subsections.
3. *Filling gaps of scaffolds:* Finally, nanoGapFiller fills the gaps of scaffolds using the nucleic base sequence of the best-matching contig paths. For example, the gap shown in Fig. 4 is filled using the best-matching contig path $c_1 c_3 c_6 c_{10}$. After filling the gaps of scaffolds, the genome completeness will be greatly improved.

### Measuring the similarity between an optical map and a contig path

Consider an optical map with site sequence $x_1 \cdots x_m$ and a contig path with site sequence $s_1 \cdots s_n$. nanoGapFiller calculates the probability that the contig path generates the optical map (denoted as $S(x_1 \cdots x_m, s_1 \cdots s_n)$), and then uses this probability as similarity between them. The generating process of $x_1 \cdots x_m$ from $s_1 \cdots s_n$ is as follows: In an ideal optical mapping experiment, an enzyme recognition site $s_i$ in the contig path will be observed and recorded as a certain site $x_j$ of the optical map, which is called *matching* between sites $s_i$ and $x_j$. However, it is often the case that some recognition sites are missing (called *deletion*) whereas some extra sites are recorded in optical maps purely due to false-positive signals (called *insertion*).

To formally describe the generating process of an optical map from a contig path, we define the alignment between their site sequences. For each alignment *A* of the sites sequences $x_1 \cdots x_m$ and $s_1 \cdots s_n$, we use $S_{A}(x_1 \cdots x_m, s_1 \cdots s_n, {A})$ to denote the possibility that the generating process corresponding to this alignment occurs.

Among all possible alignments between $x_1 \cdots x_m$ and $s_1 \cdots s_n$, we identify the one with the highest score, and then use this score as the similarity between the two site sequences, i.e.,

$$\begin{aligned} S(x_1 \cdots x_m, s_1 \cdots s_n) = \max _{ A \in {\mathcal {A}} } S_A(x_1 \cdots x_m, s_1 \cdots s_n, A), \end{aligned}$$

where ${\mathcal {A}}$ denotes the set of all possible alignments of the two site sequences.

We calculate $S_{A}(x_1 \cdots x_m, s_1 \cdots s_n, {A})$ as follows: we divide the two sequences at the matching sites of *A*, and thus acquire several *matching fragment pairs*. For example, the division at the matching sites $<x_2, s_2>$ and $<s_2, x_4>$ yields three matching fragment pairs (see Fig. 3). For each matching fragment pair *p*, we calculate three scoring items, including:

1. Length difference item *LD*(*p*): In the ideal case, two matching fragments should have identical length. However, in an optical mapping experiment, the molecules are always stretched or compressed, leading to length difference of the matched fragments. To measure the length difference, we adopted the Laplace distribution as performed by Rmaps [31–33], i.e.,
  $$\begin{aligned} LD(p) = \frac{1}{2 b} \exp \left( -\frac{|d-\mu |}{b}\right) , \end{aligned}$$
  where *d* denotes the length difference of the two matching fragments in *p*, and $\mu$ and *b* denotes the mean and scale parameter of the distribution, respectively.
2. Missing sites item *M*(*p*): We used the Geometry distribution [31–33] to model the number of missing sites *m*, i.e.,
  $$\begin{aligned} M(p) = (1-q)^{M-1} q, \end{aligned}$$
  where *q* denotes the probability that an enzyme recognition site is detected by optical mapping.
3. False-positive sites item *FP*(*p*): We used Poisson distribution to model the number of false-positive sites *f*, i.e.,
  $$\begin{aligned} FP(p) = \frac{\lambda ^{f} e^{-\lambda }}{f !}, \end{aligned}$$
  where $\lambda$ represents the expected number of false positive sites.

In this study, the parameters were set according to the manually-verified alignments of optical maps and contig paths of *E. coli* as $q=0.772, \lambda =1526000, \mu =293 nt, b=500 nt$.

### Identifying the best-matching contig path of a gap

Before describing our method to identify the contig path that best matches a given gap, we first present the formulation of this problem: Let $x_1 \cdots x_m$ be the site sequence of the gap of interest. Through locating gaps, we have identified from assembly graph two sites that match the beginning site $x_1$ and the ending site $x_m$, respectively. We denote these two identified sites as $s_b$ and $s_e$. Thus, the objective is to find the contig path with site sequence $s_b\cdots s_e$ such that the score $S(x_1 \cdots x_m, s_b \cdots s_e)$ is maximized.

The basic idea of our method is probabilistic search together with search space pruning, which can be described as follows: Starting from the beginning site $x_1$, we iterate finding the best-matching sites for each site $x_i$ $(1 \le i \le m)$ through executing the following three steps:

1. *Calculating the probability of site matching:* We use a set $M[x_i]$ to hold all matching sites of $x_i$. From the first $i-1$ sites $x_1\cdots x_{i-1}$, we calculate the belief that $x_i$ matches each site $s\in M[x_i]$, denoted as $Belief(x_i = s)$. Now we perform normalization to transform the belief into probability $\Pr [x_i = s]$.
2. *Pruning the unlikely matching pairs:* To reduce the search space, we remove the unlikely matching sites, i.e., deleting the site *s* from $M[x_i]$ if $\Pr [x_i = s]$ is less than a pre-defined threshold MinimalMatchingProbability. We will show experimental results that when setting appropriate threshold, the search space could be significantly reduced with little influence on finding the correct paths.
3. *Propagating the matching probability to downstream site-pairs:* For the left-over sites $s\in M[x_i]$, we propagate their matching probability $\Pr [x_i = s]$ to the downstream site pair $<x_j, s'>$, where $j \le i +$ MaxInsertionSize and $s'$ is within at most MaxDeletionSites from *s*. For each pair $<x_j, s'>$, we calculate its matching belief according to Bayesian formula, which uses $\Pr [x_i = s]$ as prior probability and the similarity $S(x_i\cdots x_j, s\cdots s')$ as conditional probability.

**Fig. 5:**
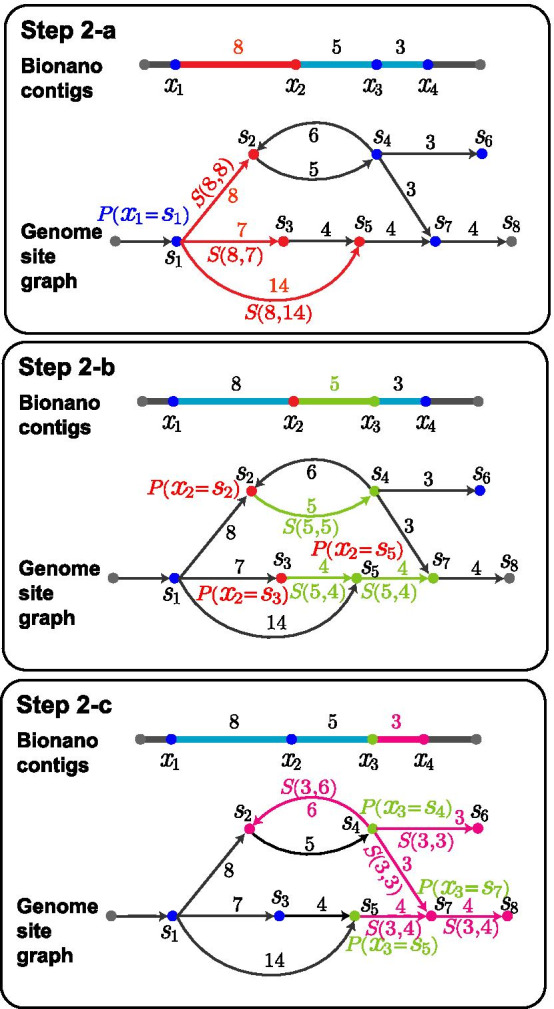
Searching site graph for the site sequence that best matches a gap. In this example, the gap has site sequence $x_1x_2x_3x_4$ with distance 8, 5, 3, respectively. Through locating gaps in Step 1, we have known that the beginning site $x_1$ matches $s_1$, and the ending site $x_4$ matches $s_7$. Thus, our objective is to find the path from $s_1$ to $s_7$ that best matches the gap $x_1x_2x_3x_4$. **a** Initially, we set $\Pr [x_1 = s_1] = 1$ as we have known $x_1$ matches $s_1$. Next we propagated this probability to downstream site pairs and calculated the following matching beliefs for site $x_2$: $Belief(x_2 = s_2) = \Pr [x_1 = s_1] S(8, 8)$, $Belief(x_2 = s_3) = \Pr [x_1 = s_1] S(8, 7)$, and $Belief(x_2 = s_5) = \Pr [x_1 = s_1] S(8, 14)$. After normalization, we obtained the site matching probabilities: $\Pr [x_2 = x_2] = 0.81$, $\Pr [x_2 = x_3] = 0.19$, and $\Pr [x_2 = x_5] = 0$. **b ** We propagated these probabilities further and obtained the following beliefs for site $x_3$ $Belief(x_3 = s_4) = \Pr [x_2=s_2] S(5, 5)$, $Belief(x_3 = s_5) = \Pr [x_2=s_3] S(5, 4)$, and $Belief(x_3 = s_7) = \Pr [x_2=s_5] S(5, 4)$ and then normalized them into probabilities. After normalization, we obtained the site matching probabilities: $\Pr [x_3 = x_4] = 0.95$, $\Pr [x_3 = x_5] = 0.05$, and $\Pr [x_3 = x_7] = 0$. **c ** For site $x_4$, we calculated its matching beliefs similarly. Note that there are two paths reaching site $s_7$, and thus we needed to calculate the maximum of the two paths as follows: $Belief(x_4 = s_7) =$ $\max \{\Pr [x_3=s_4]S(3,3),$ $\Pr [x_3 = s_5] S(3,4)\}$. After calculating $\Pr [x_4=s_8]$, we traced back and reported the best matching site path as $s_1s_2s_4s_7$

We iterate this matching site finding procedure until reaching the ending site $x_m$. Finally, we traceback from $x_m$ to identify the path matching best with the site sequence of the gap. Figure 5 shows an example of this probabilistic search procedure. The pseudocode is presented as follows. 
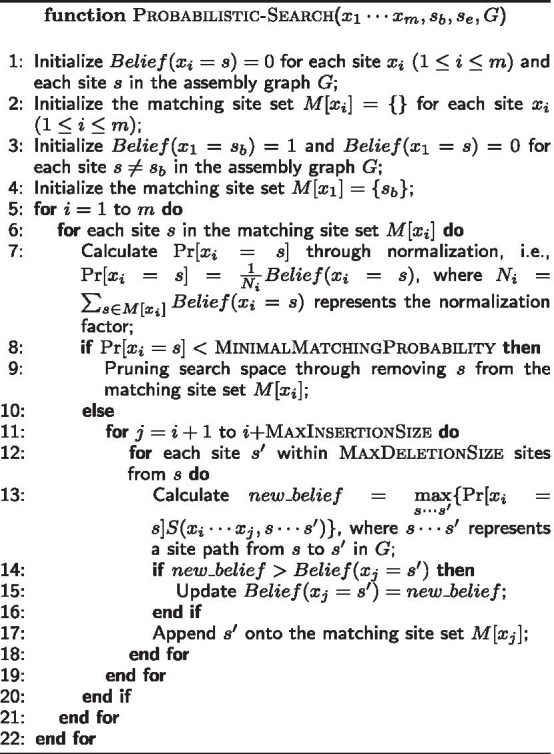

## Supplementary Information

**Additional file 1.** The additional results on the performance of nanoGapFiller.

## Data Availability

The genome reference analysed during the current study are available in the NCBI repository under access id: NC_005070.1, NC_004116.1, NC_007005.1, NC_006361.1, NC_004547.2, NC_008255.1, NC_006350.1, NC_004463.1, NC_007413.1, AL645882.2, NC_000913.2, AP013070.1. The Hi-C data are available in the NCBI GEO repository under access id: GSM2870416, GSM2870417. The optical map that support the findings of this study are available from Xuan Li but restrictions apply to the availability of these data, which were used under license for the current study, and so are not publicly available. Please contact Xuan Li (lixuan@sippe.ac.cn) if you need access these data. Source code of nanoGapFiller is freely available through https://github.com/bigict/nanoGapFiller.

## Abbreviations

DFS: Depth-first search
NGS: Next-generation sequence
CPS: Contig path similarity
NSS: Nucleotide sequence similarity
