# Supplementary material for: Filling gaps of genome scaffolds via probabilistic searching optical maps against assembly graph
Source: BMC Bioinformatics. 2021 Oct 30;22:533. doi: 10.1186/s12859-021-04448-2 (PMC8557617; doi:10.1186/s12859-021-04448-2)
Supplement: Supplementary file 1 — Additional file 1. The additional results on the performance of nanoGapFiller. [file 12859_2021_4448_MOESM1_ESM.pdf]

Filling gaps of genome scaffolds via probabilistic searching optical maps against assembly graph

Supplementary Material

Bin Huang[1,2], Guozheng Wei[1,2], Bing Wang[1,2], Fusong Ju[1,2], Yi Zhong[3], Shiwei Sun[1,2] and Dongbo Bu[1,2,*]

[1]Key Lab of Intelligent Information Processing, Big-data Academy, Institute of Computing Technology, Chinese Academy of Sciences, Beijing, 100190, China

[2]University of Chinese Academy of Sciences, Beijing, 100049, China and

[3]School of Computer Science, University of Washington, Seattle, 98195, United States

[*]To whom correspondence should be addressed.

Table of contents

(1) Supplementary Figure 1: Venn graphs of the gaps filled by nanoGapFiller and OMACC for 12 species

(2) Supplementary Table 1: Filling the gaps identified using simulated optical maps of *A. vari* genome (Alignment method: SOMA2)

(3) Supplementary Table 2: Filling the gaps identified using simulated optical maps of *B. japonicumgenome* (Alignment method: SOMA2)

(4) Supplementary Table 3: Filling the gaps identified using simulated optical maps of *B. pseudomallei* (Alignment method: SOMA2)

(5) Supplementary Table 4: Filling the gaps identified using simulated optical maps of *C. hutchinsonii* (Alignment method: SOMA2)

(6) Supplementary Table 5: Filling the gaps identified using simulated optical maps of *E. carotovora* (Alignment method: SOMA2)

(7) Supplementary Table 6: Filling the gaps identified using simulated optical maps of *N. farcinica* (Alignment method: SOMA2)

(8) Supplementary Table 7: Filling the gaps identified using simulated optical maps of *P. putida* (Alignment method: SOMA2)

(9) Supplementary Table 8: Filling the gaps identified using simulated optical maps of *P. syringae* (Alignment method: SOMA2)

(10) Supplementary Table 9: Filling the gaps identified using simulated optical maps of *S. agal* (Alignment method: SOMA2)

(11) Supplementary Table 10: Filling the gaps identified using simulated optical maps of *S. coelicolor* (Alignment method: SOMA2)

(12) Supplementary Table 11: Filling the gaps identified using simulated optical maps of *S. ynec* (Alignment method: SOMA2)

(13) Supplementary Table 12: Filling the gaps identified using real optical maps of *P. putida* (Alignment method: SOMA2)

(14) Supplementary Table 13: Filling the gaps identified using real optical maps of *S. coelicolor* (Alignment method: SOMA2)

(15) Supplementary Table 14: Genome completeness improvement after filling gaps using OMACC, nanoGapFiller and Novo&Stitch on E. coli.

(16) Supplementary Table 15: Genome completeness improvement after filling gaps using OMACC, nanoGapFiller and Novo&Stitch on P.putida.

(17) Supplementary Table 16: Genome completeness improvement after filling gaps using OMACC, nanoGapFiller and Novo&Stitch on S.coelicolor.

(18) Supplementary Table 17: Filling the gaps identified using simulated optical maps of E. coligenome. Alignment method: SOMA2.

(19) Supplementary Table 18: Filling the gaps identified using simulated optical maps of E. coligenome. Alignment method: OMBlast.

(20) Supplementary Table 19: Filling the gaps identified using simulated optical maps of E. coligenome. Simulator of optical maps: OMSim;Alignment method: refAligner.

(21) Supplementary Table 20: Scaffolding using Bionano optical mapping and Hi-C data on E. coli.

1 Supplementary Figures

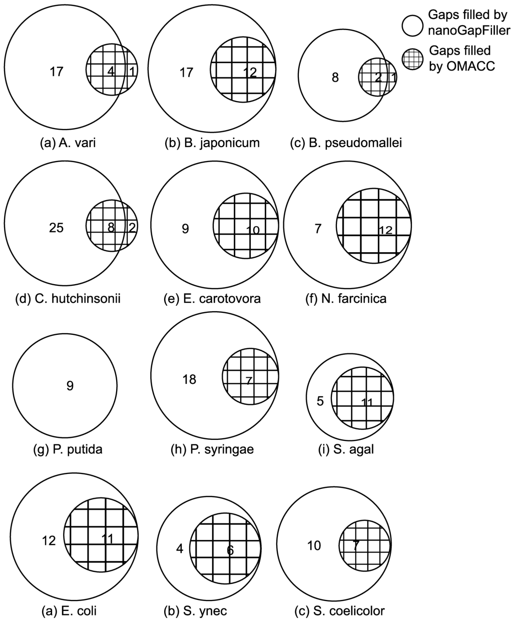

Figure 1: Venn graphs of the gaps filled by nanoGapFiller and OMACC for 12 species. Here, the gaps are identified using simulated optical maps with alignment method SOMA2

2 Supplementary Tables

Table 1: Filling the gaps identified using simulated optical maps of *A. vari* genome. Alignment method: SOMA2

	Reference sequence		OMACC				nanoGapFiller			
Gap	#contigs	#bases	#contigs	#bases	CPS	NSS	#contigs	#bases	CPS	NSS
212836-213204r	3	1090	3	1090	3	100%	3	1090	3	100%
212976r-213236	3	6418	3	6418	3	100%	3	6418	3	100%
213236-212968r	5	20266	7	20170	5	99.76%	-	-	-	
213236-212968r	7	20266	-	-	-	-	7	20266	7	100%
212904-212972r	11	58	11	58	11	100%	11	58	11	100%
212914r-213200r	12	1762	-	-	-	-	12	1762	12	100%
211666-212912r	13	38300	-	-	-	-	13	38300	13	100%
213204r-212914r	22	83829	22	83829	22	100%	22	83829	22	100%
212912r-213196r	29	207103	-	-	-	-	29	207103	29	100%
212968r-213066	33	113428	-	-	-	-	33	113428	33	100%
213118r-211666	46	287907	-	-	-	-	46	287907	46	100%
213200r-212904	67	88672	-	-	-	-	84	88564	54	99.87%
212930-213118r	67	200664	-	-	-	-	67	200664	67	100%
213066-213104	68	466075	-	-	-	-	70	466075	66	99.97%
213196r-213084r	73	433919	-	-	-	-	77	433919	71	100%
213104-213220	107	736403	-	-	-	-	107	736403	107	100%
213220-212930	133	560840	-	-	-	-	135	560849	120	99.92%

Table 2: Filling the gaps identified using simulated optical maps of *B. japonicumgenome*. Alignment method: SOMA2

	Reference sequence		OMACC				nanoGapFiller			
Gap	#contigs	#bases	#contigs	#bases	CPS	NSS	#contigs	#bases	CPS	NSS
289290r-289836r	3	552	-	-	-	-	3	552	3	100%
289760-289202r	3	1	3	1	3	100%	3	1	3	100%
289154-289764	3	1189	3	1189	3	100%	3	1189	3	100%
289748r-289306	3	4	3	4	3	100%	3	4	3	100%
289422r-289154	4	5	4	5	4	100%	4	5	4	100%
289306-289740	5	2193	5	2193	5	100%	5	2193	5	100%
289202r-289756r	5	30575	5	30575	5	100%	5	30575	5	100%
289624r-289744	5	616	5	616	5	100%	5	616	5	100%
289756r-289732	5	184	15	829	5	36.33%	5	184	5	100%
289804-289760	6	8031	6	8031	6	100%	6	8031	6	100%
289800-289596r	7	8478	7	8478	7	100%	7	8478	7	100%
289812r-289100	8	13727	-	-	-	-	8	13727	8	100%
289664-289800	9	349	-	-	-	-	10	349	7	96.28%
289584r-289628r	9	2575	-	-	-	-	9	2575	9	100%
289576-280346r	11	3575	-	-	-	-	11	3575	10	99.78%
289588-289648r	11	139	16	8187	11	3.34%	11	139	11	100%
289378r-289584r	12	1049	-	-	-	-	12	1049	9	99.81%
289752-289664	12	387	-	-	-	-	12	387	12	100%
289764-289816r	12	37981	12	37981	12	100%	12	37981	12	100%
289390-289688r	14	36867	-	-	-	-	14	36867	12	99.41%
289672-289378r	14	376	-	-	-	-	14	376	14	100%
289816r-289290r	15	636	-	-	-	-	15	636	15	100%
289768r-289346r	17	1290	-	-	-	-	17	1290	17	100%
289346r-289588	18	48850	-	-	-	-	18	48850	18	100%
289628r-289792r	20	24922	-	-	-	-	20	24922	20	100%
289740-289768r	26	8742	-	-	-	-	26	8742	23	99.98%
289830r-289672	33	28536	-	-	-	-	33	28536	31	100%
289272-289752	35	311442	-	-	-	-	36	311442	31	99.96%
289792r-289624r	40	40606	-	-	-	-	71	40213	36	98.84%

Table 3: Filling the gaps identified using simulated optical maps of *B. pseudomallei*. Alignment method: SOMA2

	Reference sequence		OMACC				nanoGapFiller			
Gap	#contigs	#bases	#contigs	#bases	CPS	NSS	#contigs	#bases	CPS	NSS
161240r-160960r	5	607	5	607	5	100%	5	607	5	100%
160676r-161188	7	5966	-	-	-	-	7	5966	7	100%
161132r-160920r	7	21609	-	-	-	-	7	21609	7	100%
161316r-160940	9	30	39	150	9	33.33%	9	30	9	100%
160920r-161316r	11	8635	-	-	-	-	17	8614	11	99.88%
161216r-160868	12	32827	-	-	-	-	12	32827	12	100%
160960r-161288	17	13454	-	-	-	-	45	13342	17	99.58%
160920r-161316r	17	8635	73	8831	17	98.88%	-	-	-	
160636r-160924	17	1124	-	-	-	-	18	1124	14	87.19%

Table 4: Filling the gaps identified using simulated optical maps of *C. hutchinsonii*. Alignment method: SOMA2

	Reference sequence		OMACC				nanoGapFiller			
Gap	#contigs	#bases	#contigs	#bases	CPS	NSS	#contigs	#bases	CPS	NSS
118744r-118632	5	182	5	182	5	100%	5	182	5	100%
118752-118690r	5	1242	5	1242	5	100%	5	1242	5	100%
118870r-118884	5	359	17	1715	5	34.62%	5	359	5	100%
119024-118856	5	251	5	251	5	100%	5	251	5	100%
118772r-118870r	5	589	5	589	5	100%	5	589	5	100%
118886r-118816r	6	5215	-	-	-	-	6	5215	6	100%
118830r-118818	6	5113	-	-	-	-	6	5113	6	100%
118632-118916r	6	1248	6	1248	6	100%	6	1248	6	100%
118856-118882	7	21605	7	21605	7	100%	7	21605	7	100%
118816r-118772r	7	5490	-	-	-	-	7	5490	7	100%
118868-119012r	11	48069	-	-	-	-	11	48069	11	100%
119012r-119028	11	11	83	95	11	20.75%	11	11	11	100%
118916r-118814r	11	6785	-	-	-	-	11	6785	8	80.53%
118848-119050r	13	24701	-	-	-	-	13	24701	13	100%
119028-118922r	13	35611	-	-	-	-	13	35611	13	100%
118922r-118848	15	35032	-	-	-	-	15	35032	13	99.84%
118892r-118744r	21	120145	-	-	-	-	21	120145	18	99.97%
118884-118868	23	2066	-	-	-	-	24	2066	22	99.90%
119050r-119020r	24	143	-	-	-	-	120	31	24	35.63%
118818-119054r	25	2470	-	-	-	-	43	2449	25	99.57%
118994-118830r	26	50	-	-	-	-	38	36	26	83.72%
119032-118836r	31	24280	-	-	-	-	33	24280	25	99.09%
118836r-118994	33	3220	-	-	-	-	36	3220	23	99.57%
118994-118830r	38	50	104	127	38	56.50%	-	-	-	
118882-118892r	51	17533	-	-	-	-	56	17533	34	96.95%
118802-119024	77	198646	-	-	-	-	78	198646	76	99.99%
119050r-119020r	120	143	120	143	120	100%	-	-	-	

Table 5: Filling the gaps identified using simulated optical maps of *E. carotovora*. Alignment method: SOMA2

	Reference sequence		OMACC				nanoGapFiller			
Gap	#contigs	#bases	#contigs	#bases	CPS	NSS	#contigs	#bases	CPS	NSS
141756r-141782	3	5	3	5	3	100%	3	5	3	100%
141782-140860	3	42	3	42	3	100%	3	42	3	100%
141760-141234r	3	20	3	20	3	100%	3	20	3	100%
141454-141904	3	1	3	1	3	100%	3	1	3	100%
141916r-141268	3	20	3	20	3	100%	3	20	3	100%
141764r-141788r	5	2897	5	2897	5	100%	5	2897	5	100%
141664r-141928	5	35374	5	35374	5	100%	5	35374	5	100%
141896-141766	5	246	5	246	5	100%	5	246	5	100%
141488-141664r	5	905	5	905	5	100%	5	905	5	100%
141718r-141586	6	14	-	-	-	-	6	14	6	100%
140860-141774r	7	27153	-	-	-	-	7	27153	7	100%
141930r-141454	7	9208	9	11362	7	89.53%	7	9208	7	100%
141554r-141916r	8	2314	-	-	-	-	8	2314	8	100%
141870r-141554r	9	13430	-	-	-	-	9	13430	8	99.96%
141774r-141476	10	3383	-	-	-	-	11	3383	9	98.14%
141876r-141760	17	23490	-	-	-	-	17	23490	16	99.94%
141268-141930r	25	103161	-	-	-	-	37	103125	25	99.98%
141768-141506r	41	81230	-	-	-	-	41	81230	30	99.91%
141766-141876r	44	70524	-	-	-	-	46	70524	42	99.99%

Table 6: Filling the gaps identified using simulated optical maps of *N. farcinica*. Alignment method: SOMA2

	Reference sequence		OMACC				nanoGapFiller			
Gap	#contigs	#bases	#contigs	#bases	CPS	NSS	#contigs	#bases	CPS	NSS
212338-212474r	3	614	3	614	3	100%	3	614	3	100%
212634r-212510	3	1035	3	1035	3	100%	3	1035	3	100%
212394r-212602	3	201	3	201	3	100%	3	201	3	100%
212566-212478	3	201	3	201	3	100%	3	201	3	100%
212478-212276	3	10	3	10	3	100%	3	10	3	100%
212618r-212634r	5	326	-	-	-	-	5	326	4	95.40%
212314r-212470r	5	784	-	-	-	-	5	784	5	100%
212108r-212394r	5	326	-	-	-	-	5	326	5	100%
212562-212092	5	771	5	771	5	100%	5	771	5	100%
212422r-212562	5	1416	7	2789	5	67.35%	5	1416	5	100%
212494r-212566	5	23	35	293	5	14.56%	5	23	5	100%
212602-212598	5	53	7	101	5	68.83%	5	53	5	100%
212474r-212108r	5	16053	5	16053	5	100%	5	16053	5	100%
212510-212338	7	189	11	339	7	71.59%	7	189	7	100%
212294-212494r	9	8364	-	-	-	-	9	8364	9	100%
212276-212554r	9	60910	11	62234	9	98.92%	9	60910	9	100%
212598-212294	14	972	-	-	-	-	15	972	11	99.59%
212286r-212314r	18	17027	-	-	-	-	18	17027	14	99.97%
212554r-212286r	25	88032	-	-	-	-	26	88032	24	100%

Table 7: Filling the gaps identified using simulated optical maps of *P. putida*. Alignment method: SOMA2

	Reference sequence		OMACC				nanoGapFiller			
Gap	#contigs	#bases	#contigs	#bases	CPS	NSS	#contigs	#bases	CPS	NSS
443842r-443858	18	1121	-	-	-	-	19	1121	13	99.55%
443918r-443826	23	112411	-	-	-	-	23	112411	21	100%
443914-443944	26	187405	-	-	-	-	33	187405	23	99.92%
443834r-443898r	31	73095	-	-	-	-	34	73083	27	99.99%
443898r-443842r	51	273141	-	-	-	-	51	273141	51	100%
443762r-443914	61	271332	-	-	-	-	63	271332	56	99.99%
443894-443762r	63	679034	-	-	-	-	66	679034	57	100%
443886-443918r	81	409883	-	-	-	-	83	409883	62	99.96%
443858-443894	112	726612	-	-	-	-	113	726612	101	99.99%

Table 8: Filling the gaps identified using simulated optical maps of *P. syringae*. Alignment method: SOMA2

Gap	Reference sequence		OMACC				nanoGapFiller			
	#contigs	#bases	#contigs	#bases	CPS	NSS	#contigs	#bases	CPS	NSS
185472r-185248r	3	202	3	202	3	100%	3	202	3	100%
185476r-185416	4	1356	4	1356	4	100%	4	1356	4	100%
185392r-185244r	5	1904	5	1904	5	100%	5	1904	5	100%
184992-184668	5	167	5	167	5	100%	5	167	5	100%
185154r-184814	5	311	5	311	5	100%	5	311	5	100%
185126r-184852	7	1628	-	-	-	-	7	1628	7	100%
184748-185472r	8	1412	8	1412	8	100%	8	1412	8	100%
185044-185058	9	17318	-	-	-	-	15	17318	7	99.98%
184878r-184918r	10	5768	-	-	-	-	10	5768	8	99.95%
185432-185190r	11	2542	-	-	-	-	11	2542	11	100%
185090r-185392r	13	18075	-	-	-	-	13	18075	6	68.82%
185150-184748	13	22492	-	-	-	-	13	22492	11	99.95%
184918r-185448r	13	1654	-	-	-	-	17	1654	9	99.76%
184974r-185044	15	159	15	159	15	100%	15	159	15	100%
184966-184992	16	21188	-	-	-	-	20	21188	14	99.79%
185420r-185126r	16	35913	-	-	-	-	16	35913	14	99.91%
184814-185432	20	8721	-	-	-	-	20	8721	20	100%
184668-185142	38	31194	-	-	-	-	38	31194	38	100%
185210-185444	39	104002	-	-	-	-	39	104002	39	100%
185244r-185396	41	19777	-	-	-	-	52	19772	27	98.03%
185396-185170r	41	951	-	-	-	-	52	951	26	96.32%
185248r-185154r	42	13131	-	-	-	-	48	13131	32	99.75%
185142-184954r	48	31163	-	-	-	-	64	31124	40	92.71%
185448r-184966	51	81685	-	-	-	-	59	81685	49	100%
185484-185476r	91	87343	-	-	-	-	93	87343	75	99.58%

Table 9: Filling the gaps identified using simulated optical maps of *S. agal* . Alignment method: SOMA2

Gap	Reference sequence		OMACC				nanoGapFiller			
	#contigs	#bases	#contigs	#bases	CPS	NSS	#contigs	#bases	CPS	NSS
54166r-54190r	3	109	3	109	3	100%	3	109	3	100%
54154-54040	3	1062	3	1062	3	100%	3	1062	3	100%
53952-53836r	3	1357	3	1357	3	100%	3	1357	3	100%
54142-54168r	5	679	5	679	5	100%	5	679	5	100%
53836r-54154	5	313	7	606	5	68.12%	5	313	5	100%
54180r-54212r	5	1241	5	1241	5	100%	5	1241	5	100%
54190r-54138r	5	1465	5	1465	5	100%	5	1465	5	100%
54176r-54022r	6	5182	6	5182	6	100%	6	5182	6	100%
54172r-54180r	6	1242	6	1242	6	100%	6	1242	6	100%
54212r-53952	10	21396	10	21396	10	100%	10	21396	10	100%
54040-54142	11	27482	11	27482	11	100%	11	27482	11	100%
54208r-54152	12	51451	-	-	-	-	12	51451	12	100%
54116r-54208r	13	6355	-	-	-	-	13	6355	13	100%
54022r-54182	20	34068	-	-	-	-	40	31698	20	96.40%
54152-54176r	20	19366	-	-	-	-	20	19366	20	100%
54182-54172r	22	71941	-	-	-	-	22	71941	22	100%

Table 10: Filling the gaps identified using simulated optical maps of *S. coelicolor*. Alignment method: SOMA2

	Reference sequence		OMACC				nanoGapFiller			
Gap	#contigs	#bases	#contigs	#bases	CPS	NSS	#contigs	#bases	CPS	NSS
781194-781794r	3	2454	3	2454	3	100%	3	2454	3	100%
781742r-781810r	3	1	-	-	-	-	3	1	3	100%
780734-781506r	3	2771	3	2771	3	100%	3	2771	3	100%
781982r-781058r	3	9	3	9	3	100%	3	9	3	100%
781988-780466r	5	1224	-	-	-	-	5	1224	5	100%
782030-781742r	5	1476	5	1476	5	100%	5	1476	5	100%
781730-782030	5	5897	15	8101	5	84.25%	5	5897	5	100%
781206r-781916	7	2711	-	-	-	-	7	2711	6	98.23%
781570r-781558r	11	27980	-	-	-	-	11	27980	9	99.06%
781794r-781686r	11	10158	-	-	-	-	11	10158	11	100%
781314-781976	14	1178	-	-	-	-	14	1178	14	100%
781916-781798r	14	23647	30	24583	14	98.06%	14	23647	14	100%
781686r-782078r	15	6089	-	-	-	-	15	6089	15	100%
781940r-782066r	16	11886	16	11886	16	100%	16	11886	16	100%
781506r-781414	23	85226	-	-	-	-	23	85226	23	100%
781964-781450r	42	33991	-	-	-	-	43	33991	34	92.34%
781442r-782042	58	51998	-	-	-	-	63	51998	47	99.41%

Table 11: Filling the gaps identified using simulated optical maps of S. ynec. Alignment method: SOMA2

	Reference sequence		OMACC				nanoGapFiller			
Gap	#contigs	#bases	#contigs	#bases	CPS	NSS	#contigs	#bases	CPS	NSS
63852-63934	3	5936	-	-	-	-	3	5936	3	100%
63258-63852	3	42	3	42	3	100%	3	42	3	100%
63856r-63878r	3	966	3	966	3	100%	3	966	3	100%
63886r-63876	5	4499	5	4499	5	100%	5	4499	5	100%
63808-63886r	5	3755	-	-	-	-	5	3755	5	100%
63876-63898	5	528	-	-	-	-	5	528	4	95.64%
63862-63904	7	70	7	70	7	100%	7	70	7	100%
63878r-63854	7	9800	7	9800	7	100%	7	9800	7	100%
63870r-63808	7	3692	7	3692	7	100%	7	3692	7	100%
63936r-63258	9	1412	-	-	-	-	9	1412	9	100%

Table 12: Filling the gaps identified using real optical maps of *P. putida*. Alignment method: SOMA2

	Reference sequence		OMACC				nanoGapFiller			
Gap	#contigs	#bases	#contigs	#bases	CPS	NSS	#contigs	#bases	CPS	NSS
442956r-443944r	3	9	3	9	3	100%	3	9	3	100%
443944r-443300r	6	40057	6	40057	6	100%	6	40057	6	100%
443914r-443710	7	5266	-	-	-	-	7	5266	7	100%

Table 13: Filling the gaps identified using real optical maps of *S. coelicolor*. Alignment method: SOMA2

	Reference sequence		OMACC				nanoGapFiller			
Gap	#contigs	#bases	#contigs	#bases	CPS	NSS	#contigs	#bases	CPS	NSS
781414r-781790r	8	9948	-	-	-	-	8	9948	8	100%

Table 14: Genome completeness improvement after filling gaps using OMACC, nanoGapFiller and Novo&Stitch on *E. coli*. Here, the original assembly results are generated using SPAdes on simulated sequencing reads. The assembly results are evaluated using Quast

		After gap filling		
	Before gap filling	OMACC	nanoGapFiller	Novo&Stitch
# contigs ($\geq$ 0 bp)	376	314	139	9
# contigs ($\geq$ 1000 bp)	113	94	54	9
# contigs ($\geq$ 5000 bp)	84	68	41	9
# contigs ($\geq$ 10000 bp)	75	60	35	9
# contigs ($\geq$ 25000 bp)	57	44	24	9
# contigs ($\geq$ 50000 bp)	31	26	13	9
Total length ($\geq$ 0 bp)	4591124	4594237	**4610590**	1517294
Total length ($\geq$ 1000 bp)	4543853	4554461	**4596046**	1517294
Total length ($\geq$ 5000 bp)	4477037	4491628	**4558525**	1517294
Total length ($\geq$ 10000 bp)	4405980	4428670	**4510884**	1517294
Total length ($\geq$ 25000 bp)	4114402	4167709	**4328110**	1517294
Total length ($\geq$ 50000 bp)	3183019	3556147	**3956200**	1517294
# contigs	125	101	56	9
Largest contig	327107	548365	**894614**	327107
Total length	4552054	4559496	**4597570**	1517294
Reference length	4639675	4639675	4639675	4639675
GC (%)	50.75	50.76	50.78	51.13
Reference GC (%)	50.79	50.79	50.79	50.79
N50	78648	133054	**785645**	166161
NG50	78648	133054	**785645**	-
N75	42010	57880	**156843**	140985
NG75	41323	57842	**156843**	-
L50	17	9	**3**	4
LG50	17	9	**3**	-
L75	37	24	7	**6**
LG75	38	25	**7**	-
# misassemblies	**0**	2	**0**	**0**
# misassembled contigs	**0**	1	**0**	**0**
Misassembled contigs length	**0**	83834	**0**	**0**
# local misassemblies	**0**	19	**0**	**0**
# unaligned contigs	0 + 0 part	0 + 0 part	0 + 0 part	0 + 0 part
Unaligned length	0	0	0	0
Genome fraction (%)	98.071	98.142	**99.065**	32.703
Duplication ratio	**1.000**	1.001	**1.000**	**1.000**
# N's per 100 kbp	0.00	0.00	0.00	0.00
# mismatches per 100 kbp	0.29	0.59	9.40	**0.00**
# indels per 100 kbp	0.02	0.29	0.78	**0.00**
Largest alignment	327107	548284	**894614**	327107
Total aligned length	4552054	4558857	**4597570**	1517294
NA50	78648	133054	**785645**	166161
NGA50	78648	133054	**785645**	-
NA75	42010	53646	**156843**	140985
NGA75	41323	46196	**156843**	-
LA50	17	9	**3**	4
LGA50	17	9	**3**	-
LA75	37	25	7	**6**
LGA75	38	26	**7**	-

Table 15: Genome completeness improvement after filling gaps using OMACC, nanoGapFiller and Novo&Stitch on *P. putida*. Here, the original assembly results are generated using SPAdes on simulated sequencing reads. The assembly results are evaluated using Quast

		After gap filling		
	Before gap filling	OMACC	nanoGapFiller	Novo&Stitch
# contigs (≥ 0 bp)	376	376	147	17
# contigs (≥ 1000 bp)	96	96	25	17
# contigs (≥ 5000 bp)	73	73	22	17
# contigs (≥ 10000 bp)	69	69	21	17
# contigs (≥ 25000 bp)	52	52	17	17
# contigs (≥ 50000 bp)	40	40	11	17
Total length (≥ 0 bp)	6108858	6108858	**6141718**	3129046
Total length (≥ 1000 bp)	6055446	6055446	**6118351**	3129046
Total length (≥ 5000 bp)	6007997	6007997	**6111222**	3129046
Total length (≥ 10000 bp)	5980013	5980013	**6102882**	3129046
Total length (≥ 25000 bp)	5709560	5709560	**6043577**	3129046
Total length (≥ 50000 bp)	5275718	5275718	**5830177**	3129046
# contigs	110	110	28	17
Largest contig	261129	261129	**3749423**	261129
Total length	6065027	6065027	**6120564**	3129046
Reference length	6156701	6156701	6156701	6156701
GC (%)	62.41	62.41	62.37	62.42
Reference GC (%)	62.33	62.33	62.33	62.33
N50	127879	127879	**3749423**	202711
NG50	127879	127879	**3749423**	107169
N75	82003	82003	**1156056**	154105
NG75	81811	81811	**1156056**	-
L50	16	16	**1**	7
LG50	16	16	**1**	17
L75	30	30	**2**	12
LG75	31	31	**2**	-
# misassemblies	0	0	0	0
# misassembled contigs	0	0	0	0
Misassembled contigs length	0	0	0	0
# local misassemblies	**0**	**0**	1	**0**
# unaligned contigs	0 + 0 part	0 + 0 part	0 + 0 part	0 + 0 part
Unaligned length	0	0	0	0
Genome fraction (%)	98.473	98.473	**99.383**	50.823
Duplication ratio	1.000	1.000	1.000	1.000
# N's per 100 kbp	0.00	0.00	0.00	0.00
# mismatches per 100 kbp	0.36	0.36	9.54	**0.03**
# indels per 100 kbp	**0.00**	**0.00**	0.42	**0.00**
Largest alignment	261129	261129	**3749423**	261129
Total aligned length	6065027	6065027	**6120318**	3129046
NA50	127879	127879	**3749423**	202711
NGA50	127879	127879	**3749423**	107169
NA75	82003	82003	**1155810**	154105
NGA75	81811	81811	**1155810**	-
LA50	16	16	**1**	7
LGA50	16	16	**1**	17
LA75	30	30	**2**	12
LGA75	31	31	**2**	-

Table 16: Genome completeness improvement after filling gaps using OMACC, nanoGapFiller and Novo&Stitch on *S. coelicolor*. Here, the original assembly results are generated using SPAdes on simulated sequencing reads. The assembly results are evaluated using Quast

		After gap filling		
	Before gap filling	OMACC	nanoGapFiller	Novo&Stitch
# contigs (≥ 0 bp)	466	422	281	20
# contigs (≥ 1000 bp)	177	162	124	20
# contigs (≥ 5000 bp)	132	122	99	20
# contigs (≥ 10000 bp)	120	110	91	20
# contigs (≥ 25000 bp)	87	79	66	20
# contigs (≥ 50000 bp)	60	54	45	20
Total length (≥ 0 bp)	8910950	**9002812**	8913948	3921657
Total length (≥ 1000 bp)	8842630	**8939647**	8877386	3921657
Total length (≥ 5000 bp)	8725868	**8836988**	8809236	3921657
Total length (≥ 10000 bp)	8639549	8750669	**8752076**	3921657
Total length (≥ 25000 bp)	8053855	8199406	**8295231**	3921657
Total length (≥ 50000 bp)	7087955	7308990	**7559270**	3921657
# contigs	210	193	140	20
Largest contig	449677	788219	**1094731**	449677
Total length	8867146	**8962557**	8889068	3921657
Reference length	9054847	9054847	9054847	9054847
GC (%)	72.08	72.12	72.07	72.52
Reference GC (%)	72.00	72.00	72.00	72.00
N50	108454	120283	201520	**230559**
NG50	107692	120270	**201520**	-
N75	61314	65092	78200	**148179**
NG75	57913	64556	**70981**	-
L50	26	20	12	**7**
LG50	27	21	**12**	-
L75	53	44	31	**13**
LG75	55	45	**33**	-
# misassemblies	**0**	2	**0**	**0**
# misassembled contigs	**0**	1	**0**	**0**
Misassembled contigs length	**0**	788219	**0**	**0**
# local misassemblies	**0**	194	3	**0**
# unaligned contigs	**0 + 0 part**	**0 + 0 part**	0 + 1 part	**0 + 0 part**
Unaligned length	**0**	**0**	1057	**0**
Genome fraction (%)	97.861	97.886	**98.102**	43.310
Duplication ratio	1.001	1.011	1.001	**1.000**
# N's per 100 kbp	0.00	0.00	0.00	0.00
# mismatches per 100 kbp	0.17	3.93	7.93	**0.00**
# indels per 100 kbp	**0.00**	1.14	1.09	**0.00**
Largest alignment	449677	550660	**1094731**	449677
Total aligned length	8867146	**8954481**	8887600	3921657
NA50	108454	120270	201520	**230559**
NGA50	107692	120270	**201520**	-
NA75	61314	65092	78200	**148179**
NGA75	57913	62901	**70981**	-
LA50	26	22	12	**7**
LGA50	27	22	**12**	-
LA75	53	45	31	**13**
LGA75	55	47	**33**	-

Table 17: Filling the gaps identified using simulated optical maps of *E. coli* genome. Alignment method: SOMA2.

	Reference sequence		nanoGapFiller			
Gap	#contigs	#bases	#contigs	#bases	CPS	NSS
127302r-127110	4	3077	4	3077	4	100.00%
127032-127424	4	8117	4	8117	4	100.00%
127304-127288r	5	27714	5	27714	5	100.00%
127230-127324r	5	10537	5	10537	5	100.00%
127314r-127304	5	38543	5	38543	5	100.00%
126832-127436r	6	9087	6	9087	6	100.00%
126702-127302r	7	15239	7	15239	7	100.00%
127058-126702	7	750	7	750	7	100.00%
127328-127332r	9	43188	9	43188	9	100.00%
127332r-127050r	10	54309	10	54309	10	100.00%
127110-126832	14	34751	14	34751	14	100.00%
127288r-127338r	15	28755	15	28755	15	100.00%
126926r-127342r	17	28060	18	28060	15	99.90%
127324r-127148r	18	17532	18	17532	14	98.95%
126836-127252	21	8563	21	8563	21	100.00%
127148r-127032	21	23780	23	23780	19	99.94%
127424-127170	23	14340	23	14340	20	99.98%
127338r-127294	23	85764	23	85764	22	99.99%
127220-127314r	26	26873	26	26873	24	99.30%
127252-127058	39	161713	39	161713	29	99.92%
127260-127230	49	93382	50	93382	43	99.92%
127170-127216r	54	129238	55	129238	51	100.00%
127334-127220	60	58953	66	58953	48	99.20%

Table 18: Filling the gaps identified using simulated optical maps of *E. coli* genome. Alignment method: OMBlast.

	Reference sequence		nanoGapFiller			
Gap	#contigs	#bases	#contigs	#bases	CPS	NSS
127338r-127256	3	18049	3	18049	3	100.00%
127342r-126680	5	10020	5	10020	5	100.00%
127314r-127304	5	38934	5	38934	5	100.00%
127058-127302r	9	54890	13	54362	9	99.48%
127332r-127050r	10	55002	10	55002	10	100.00%
127256-127294	13	26294	21	24671	13	95.75%
127304-127338r	13	69785	19	68780	13	98.97%
127216r-127332r	14	121825	32	119589	14	98.86%
126926r-127342r	16	29369	18	28412	13	97.47%
127316r-127252	16	54406	23	53629	15	99.25%
127302r-127436r	22	123508	22	123508	22	100.00%
127286r-127058	26	103134	35	101796	19	99.20%
127436r-127260	30	112925	35	112103	23	99.47%
127386r-127334	37	120078	37	120078	37	100.00%
127340r-127216r	39	70805	44	70193	26	99.34%
127148r-127340r	48	166446	56	165438	35	99.53%
127294-126926r	51	193443	55	192663	48	99.76%
126680-127316r	56	271072	65	268878	48	96.97%
127334-127314r	61	125409	91	119296	49	97.21%
127260-127148r	62	205936	71	204880	52	99.44%
127416r-127386r	74	178513	85	176884	67	99.43%

Table 19: Filling the gaps identified using simulated optical maps of *E. coli* genome. Simulator of optical maps: OMSim; Alignment method: refAligner.

	Reference sequence		nanoGapFiller			
Gap	#contigs	#bases	#contigs	#bases	CPS	NSS
252226r-252196r	15	70874	20	70252	15	99.40%
252526r-252216	30	253158	34	252630	30	99.89%
252216-252226r	31	164810	42	163227	27	99.48%
252486r-252526r	33	113104	38	112282	25	99.45%
252196r-252510r	60	335251	69	333057	52	97.58%
252312r-252486r	65	205398	71	203540	28	86.95%
252510r-252292r	86	623503	107	619061	80	99.62%
252292r-252514r	173	726650	206	720050	156	99.50%

Table 20: Scaffolding using Bionano optical mapping and Hi-C data on *E. coli*. Here, the Hi-C data are downloaded from NCBI GEO (GSM2870416, GSM2870417), and we use the software 3D-DNA for Hi-C scaffolding. As comparison, we scaffold genome using simulated optical maps and then use nanoGapFiller to fill gaps

	Before scaffolding	Optical map scaffolding and gap filling	Hi-C scaffolding
# contigs (≥ 0 bp)	376	139	16
# contigs (≥ 1000 bp)	113	54	2
# contigs (≥ 5000 bp)	84	41	2
# contigs (≥ 10000 bp)	75	35	2
# contigs (≥ 25000 bp)	57	24	2
# contigs (≥ 50000 bp)	31	13	2
Total length (≥ 0 bp)	4591124	4610590	**4637496**
Total length (≥ 1000 bp)	4543853	4596046	**4635963**
Total length (≥ 5000 bp)	4477037	4558525	**4635963**
Total length (≥ 10000 bp)	4405980	4510884	**4635963**
Total length (≥ 25000 bp)	4114402	4328110	**4635963**
Total length (≥ 50000 bp)	3183019	3956200	**4635963**
# contigs	125	56	2
Largest contig	327107	894614	**4375178**
Total length	4552054	4597570	**4635963**
Reference length	4639675	4639675	4639675
GC (%)	50.75	50.78	50.75
Reference GC (%)	50.79	50.79	50.79
N50	78648	785645	**4375178**
NG50	78648	785645	**4375178**
N75	42010	156843	**4375178**
NG75	41323	156843	**4375178**
L50	17	3	**1**
LG50	17	3	**1**
L75	37	7	**1**
LG75	38	7	**1**
# misassemblies	**0**	**0**	49
# misassembled contigs	**0**	**0**	2
Misassembled contigs length	**0**	**0**	4635963
# local misassemblies	**0**	**0**	6
# unaligned contigs	**0 + 0 part**	**0 + 0 part**	0 + 1 part
Unaligned length	**0**	**0**	2645
Genome fraction (%)	98.071	**99.065**	98.438
Duplication ratio	**1.000**	**1.000**	1.014
# N's per 100 kbp	**0.00**	**0.00**	1358.94
# mismatches per 100 kbp	**0.29**	9.40	1.71
# indels per 100 kbp	**0.02**	0.78	0.07
Largest alignment	327107	894614	**1747645**
Total aligned length	4552054	**4597570**	4569932
NA50	78648	**785645**	438708
NGA50	78648	**785645**	438708
NA75	42010	156843	**255311**
NGA75	41323	156843	**255311**
LA50	17	**3**	**3**
LGA50	17	**3**	**3**
LA75	37	7	**6**
LGA75	38	7	**6**
